# Understanding the Barriers and Attitudes toward Influenza Vaccine Uptake in the Adult General Population: A Rapid Review

**DOI:** 10.3390/vaccines11010180

**Published:** 2023-01-13

**Authors:** Verna L. Welch, Tom Metcalf, Richard Macey, Kristen Markus, Amy J. Sears, Ashley Enstone, Jakob Langer, Amit Srivastava, Alejandro Cane, Timothy L. Wiemken

**Affiliations:** 1 Pfizer Vaccines Medical & Scientific Affairs, Collegeville, PA 19426, USA; 2 Adelphi Values PROVE, Bollington SK10 5JB, UK; 3 Pfizer Patient & Health Impact, 2740-271 Lisbon, Portugal; 4 Orbital Therapeutics, Cambridge, MA 02139, USA

**Keywords:** education, uptake, barriers, promoters, strategies, hesitancy, influenza, vaccination, public health

## Abstract

Influenza is a common respiratory infection associated with a substantial clinical, humanistic, and economic burden globally. Vaccines are essential to prevent and control influenza and are recommended by public-health agencies, such as the WHO and US CDC; however, vaccination rates vary considerably across the globe. This review aimed to investigate the perceived barriers and attitudes to influenza vaccination in the global population, in order to identify strategies that may improve influenza vaccination coverage. A structured literature search was undertaken to identify studies that reported on patient-reported attitudes towards influenza vaccination, focused on the adult general population in 16 prespecified countries. Eighty studies were included in this review. Negative attitude towards healthcare were found to be the most agreed upon barrier to vaccine uptake (31.1% agreement). The most agreed promoter of influenza vaccination was trust in healthcare services (62.0% agreement). Approximately 50% of participants intended to receive the influenza vaccine in the following season. To improve influenza vaccination coverage, healthcare workers must strengthen the foundation of substantial trust in healthcare services and provide educational materials that improve influenza vaccination knowledge among the adult general population.

## 1. Introduction

Influenza is one of the most widely circulating respiratory virus infections worldwide and is associated with a substantial clinical, humanistic, and economic burden [1]. The World Health Organization (WHO) estimated in 2017 that there are one billion cases of influenza each year globally, with 3–5,000,000 cases causing severe infections [2]. Although the majority of people with influenza recover within a week without seeking medical attention, an estimated 290,000–650,000 deaths worldwide are attributable to influenza each year [3,4]. Individuals with underlying comorbid conditions, such as cardiovascular disease, chronic respiratory diseases, diabetes, obesity, neurologic conditions, and bacterial co-infections, are particularly susceptible to influenza infections, which exacerbates the overall epidemiologic burden [5,6].

Vaccines are key to the prevention and control of influenza and are recommended by several global and regional public-health agencies; however, there are conflicting recommendations between countries as to which populations should be the primary focus of influenza vaccination. The WHO recommends annual vaccination for: pregnant women, children (aged six months to five years), older adults (≥65 years of age), individuals with various comorbid conditions, and healthcare workers (HCWs) [7], whereas the US CDC recommends that all persons six months and older should receive annual influenza vaccination, and they emphasize vaccination of high-risk populations, such as adults aged ≥65 years, adults with chronic health conditions, and during pregnancy [8].

Despite such recommendations by the WHO and US CDC, influenza vaccination rates vary considerably across global regions [1]. Vaccination coverage rates among all persons aged >6 months in the last five years varied significantly from the lowest recorded rate of 11.0% in Saudi Arabia (2021) and the highest recorded vaccination coverage of 92.0% in Brazil (2018) [9]. A breakdown of influenza vaccination coverage rates in the US among the population aged >6 months revealed that the coverage rates for older adult (69.8%) and pediatric (63.8%) populations were consistently higher than the national average (51.8%), which suggests that the coverage rate for adults aged 18–64 years is substantially lower than the average rate of the total population [9]. Understanding the barriers to vaccination and drivers of vaccine uptake across regions is, therefore, pivotal to improve global influenza vaccination coverage.

The WHO does not provide a specific recommendation for influenza vaccination for the general population aged 18–64 years [7]. Only a limited number of studies are available investigating the attitudes towards influenza vaccination for all adults [10], though the need for attention to racial equity in influenza vaccination programs is well-documented [11,12,13]. This study identified differences in both attitude and vaccine coverage rates across these two sub-groups and encouraged future research on vaccine-seeking behaviors. This review aims to understand comprehensively the perceived barriers and attitudes to influenza vaccination from the individual’s perspective for the adult general population (persons aged 18–64 years and encompassing all demographics and disease states) and to identify potential strategies to overcome identified barriers to influenza vaccine uptake. 

## 2. Methods

### 2.1. Search Strategy

A structured search strategy was designed to identify studies reporting on attitudes and perceptions towards influenza vaccination in the adult general population. The search was limited to studies published between January 2012 and the start of May 2022 from pre-specified countries: France, Germany, Italy, Spain, United Kingdom (UK), United states (US), Australia, Brazil, Canada, China, Hong Kong, Japan, Mexico, Saudi Arabia, South Africa, and Taiwan. This review’s objective was to be as representative as feasible of the vast and disparate regions of interest globally. While influenza is a public-health concern in many countries, there are substantial disparities in surveillance infrastructure, testing and reporting practices, health-care services/costs, vaccination policy and implementation, and the availability of published research literature documenting these differences. The list of countries selected reflects our best attempt to balance these issues within our review strategy; in addition, the selected countries provide representation from all WHO regions.

The Cochrane guidelines for rapid reviews (outlined by Garritty et al. 2021) were followed [14]. An electronic database search was designed and conducted on 6 May 2022 in Embase and Medline via the OVID^®^ platform. The search terms included a combination of search strings comprising keywords relating to the barriers to influenza vaccine uptake (see Supplementary Material, Tables S1 and S2).

In addition, conference proceedings and gray literature were reviewed to supplemental electronic database searches. Conference proceedings were selected from a wider review of relevant conferences and chosen based on the number of relevant publications. IDWeek (a joint annual meeting of the Infectious Diseases Society of America (IDSA), Society for Healthcare Epidemiology of America (SHEA), the Society of Infectious Diseases Pharmacists (SIDP)), and the International Society for Influenza and other Respiratory Virus Diseases (ISIRV)), dating from 1 January 2020 to 6 May 2022, was included in the search. Bibliographies of relevant systematic literature reviews (SLRs) and meta-analyses identified in electronic database searches were reviewed to identify any additional relevant studies. Gray literature reporting the most recent vaccination strategies and key public-health reports from the specified countries and the WHO’s Vaccine Action Plan (global and/or regional adaptations) were also included.

### 2.2. Study Eligibility Criteria

All identified publications were screened against the Population, Interventions, Comparisons, Outcomes, Time, and Study design (PICOTS) criteria outlined in Table 1. It was expected that the focus on outcomes reported from a patient perspective would comprehensively capture the attitudes of the adult general population towards influenza vaccination. Studies that did not explicitly report outcomes from a patient perspective for the adult general population were excluded. Studies reporting on patients predominantly between the ages of 18 and 64 years but spanned across the age range (i.e., 16–69 years) were included. Studies were also included as long as they provided granular data for the age group of interest.

Studies focusing on a special population (i.e., pregnant women or HCWs) were excluded from this review as recommendations for influenza vaccination and barriers to vaccine uptake may vary from the general population.

To ensure influenza was the primary study focus, studies reporting respiratory infections, such as pneumonia or COVID-19, were only included if explicitly reported as a secondary infection to influenza. Publications focusing on attitudes toward COVID-19 vaccinations were excluded; however, publications reporting on the impact of COVID-19 on attitudes toward influenza vaccines were included.

### 2.3. Study Selection and Data Extraction

Abstract and full-text screenings were conducted by a single reviewer and quality checked by a second reviewer. Data extraction was conducted by a single reviewer and fully verified by a second reviewer. Publication information, study methods, study characteristics, population characteristics, and relevant outcomes of interest (Table 1) were extracted for each of the included studies. Data on subgroups (comorbidities, employment status, ethnicity, and income) were captured where available. A risk-of-bias assessment was performed using the JBI Critical Appraisal Checklists (Supplementary Material, Table S3) [15].

During data extraction, reviewers subjectively labelled question types using the definitions provided in Table 2. Question types were chosen based on best fit for the questionnaire item used by individual studies. Question types were further categorized to allow for more meaningful comparison across studies and to investigate the key barriers or promoters to influenza vaccine uptake. A secondary analysis was conducted to determine the agreement rate between the questions asked and the participants’ perception. Agreement rate was calculated as the percentage of participants that agreed that the barrier/promoter questioned by the study influenced their decision to be vaccinated in past or future season(s). 

## 3. Results

### 3.1. Summary of Results

**Figure 1:**
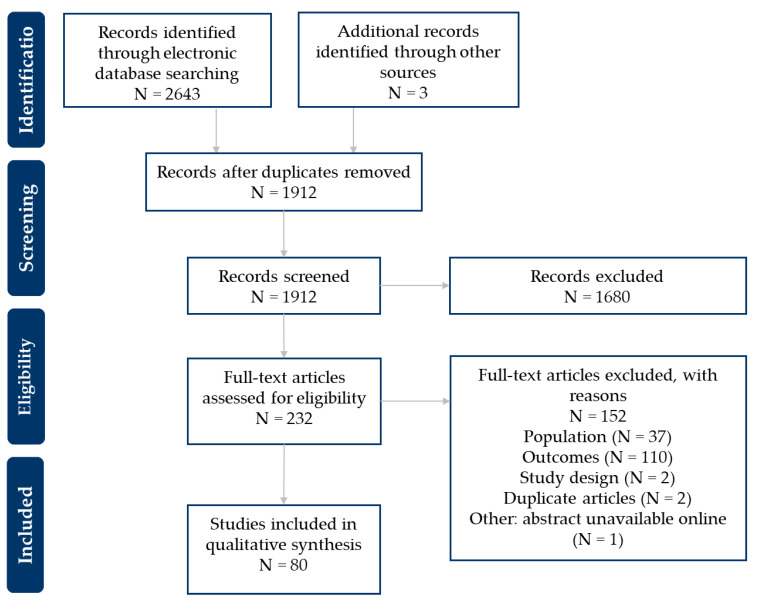
PRISMA flowchart of publications included in the review.

This structured literature review identified 80 publications via electronic databases and gray literature searches. The number of eligible publications identified during the literature searches and screening process is presented in a PRISMA flow diagram (Figure 1). Most papers (N = 110) were excluded at full-text screening for not meeting the outcome eligibility criteria. The primary reason for exclusion was not reporting barriers or attitudes from a patient’s perspective.

### 3.2. Study Characteristics

**Figure 2:**
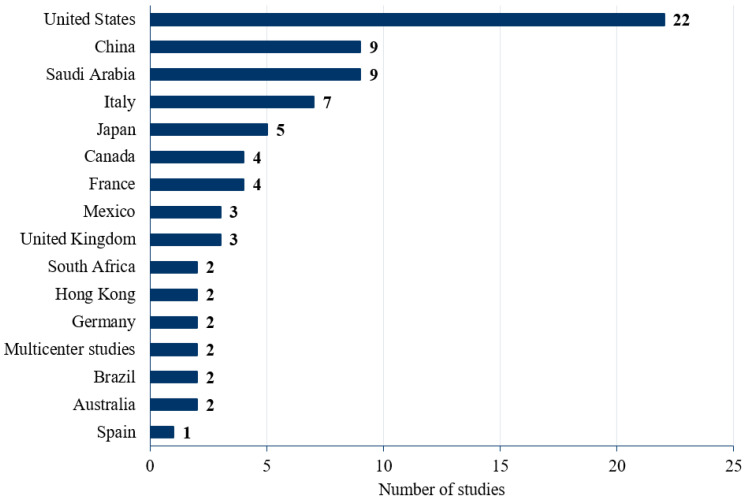
Studies reporting the barriers to influenza vaccination by country.

A breakdown of the included publications by country is presented in Figure 2. Of the 80 studies reporting on attitudes towards influenza vaccination, the majority were conducted in the US (N = 22). Thus, 61 studies presented data for the barriers to vaccination, 30 studies reported on promoters of vaccination, 34 reported patients’ intention to be vaccinated, and 64 studies suggested strategies to improve the rate of vaccination against influenza.

Cross-sectional studies were the most common study type (N = 68). The remaining studies were prospective analyses (N = 6), longitudinal (N = 4), case control (N = 1), and retrospective (N = 1). From the 80 studies included in this review, 16 reported on data collected from 2020 onward. Four longitudinal studies were captured by this review, of which only Domnich et al. 2021 investigated a change in attitudes during the COVID-19 pandemic [36].

### 3.3. Patient Characteristics

The studies captured by this review included a general population of participants aged predominantly 18–64 years. The intention of this review was to capture a wide variety of characteristics to ensure the population sample was representative of census data and, therefore, generalizable to the global population. To illustrate the variation in demographics across individual studies, a summary of participant characteristics from 22 US-based studies is presented in Table 3 [16,26,28,31,34,38,39,40,41,42,43,44,45,46,47,48,49,50,51,52,53,54]. The heterogeneity reported across US-based studies was comparable to the observed data from other countries. An implication of capturing the adult general population within this review meant substantial variation across subjects’ race, educational status, economic background, and employment status, as well as a broad range in age. However, there is limited stratification of attitudes towards vaccination by sub-population.

### 3.4. Barriers to Vaccination

Sixty-one studies reported perceived barriers to receiving an influenza vaccination in the total population (Table 4) [16,17,19,21,22,23,24,26,27,28,29,30,31,32,33,34,35,36,37,38,40,41,43,45,46,47,48,49,50,52,53,54,55,56,57,58,59,60,61,62,63,64,65,66,67,68,69,70,71,72,73,74,75,76,77,78,79,80,81,82,83,84]. Despite social factors being the most frequently investigated barriers, lack of trust was the barrier with the highest agreement rate (20.6%). The perceived barriers specific to the unvaccinated population were assessed in 20 publications. Similarly, social barriers were most commonly investigated (N = 67), although the unvaccinated population most frequently reported a perceived lack of knowledge of influenza vaccines (32.3%) [16,19,21,23,24,31,34,37,41,45,46,47,57,58,60,68,69,75,77,79].

**Figure 3:**
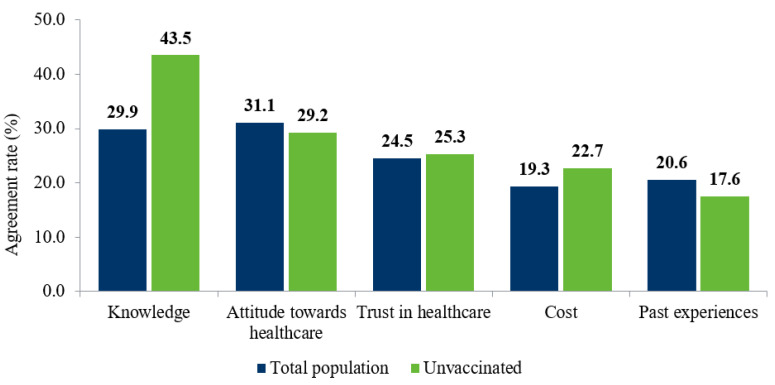
Question types as barriers to vaccination: total versus unvaccinated population.

Barrier types were further assessed to determine the key factors (determined by agreement rate) across the general and unvaccinated population for not receiving an influenza vaccine. The top-five barriers to influenza vaccination, as reported by the total population compared to the unvaccinated population, are illustrated in Figure 3 [16,17,19,21,22,23,24,26,27,28,29,30,31,32,33,34,35,36,37,38,40,41,43,45,46,47,48,49,50,52,53,54,55,56,57,58,59,60,61,62,63,64,65,66,67,68,69,70,71,72,73,74,75,76,77,78,79,80,81,82,83,84]. The most agreed upon barrier for the unvaccinated population was a perceived lack of knowledge (43.5%), whereas only 29.9% of the total population identified this as a barrier. There was little difference between the unvaccinated and total populations when comparing other barriers (Figure 3). Costs associated with influenza vaccination were the fourth-most-prevalent barrier; however, this varied due to the heterogeneity in reimbursement practices for influenza vaccines between different countries and sub-populations [21,24,27,33,39,52,67,69,73,82].

A perceived lack of knowledge of the influenza vaccine was the barrier with the highest agreement rate among the unvaccinated population. This perceived limited knowledge was further detailed by the quote “I’m swallowing, I’m injected, I’m taking it because it’s a pharmaceutical company and that’s it? What kind of oversight are we talking about here?” from a participant in the Quinn et al. 2016 trial. This unprompted response, which was typical of the questions agreed with across the included publications, further supports a lack of knowledge and trust as major barriers to vaccination. It is important to note that many participants were not opposed to the vaccine; instead, they simply did not consider influenza to be a sufficient health threat to seek out vaccination. An example of this attitude towards healthcare was stated by an unvaccinated participant in the same trial, “I think of myself as a very healthy person, it just doesn’t matter to me.” [47].

### 3.5. Promoters of Vaccination

Promoters of vaccine uptake were categorized using the definitions detailed in Table 2. Thirty studies reported data on factors the adult general population considered a promoter to vaccine uptake (Table 5) [16,17,19,20,21,22,23,24,28,31,33,35,36,38,41,46,47,52,57,61,62,63,64,67,68,71,72,75,77,85]. Social promoters were investigated most often (N = 125); however, trust in HCWs was the promoter with the highest agreement rate (68.1%). Data on promoter types for vaccinated participants were reported by 12 studies. The findings were similar to what was reported across all subjects; social factors were most commonly investigated (N = 26); however, trust in HCWs was the driving force for vaccine uptake (79.0%) [16,19,21,23,29,31,37,41,46,57,68,77]. HCWs were defined as all healthcare personnel, from doctors and pharmaceutical companies to government officials; however, none of the included publications compared trust across the differing roles played in healthcare [16,19,21,29,43].

**Figure 4:**
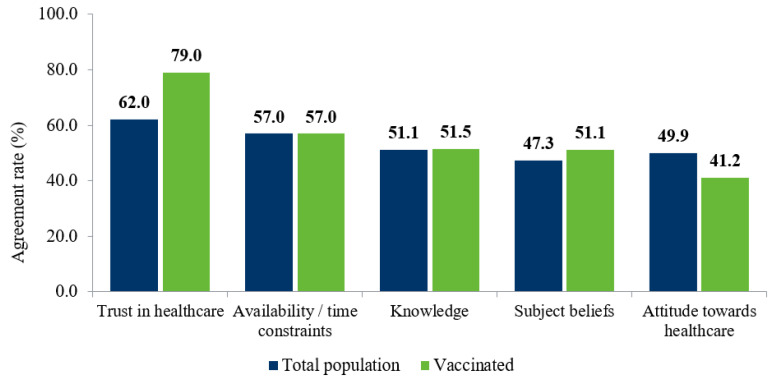
Question types as promoters of vaccination: total versus vaccinated population.

The key promoters of influenza vaccination in the adult general population and the vaccinated population were investigated. Figure 4 illustrates the top-five promoters of influenza vaccination, as reported by the total population compared to the vaccinated population. A slightly lower proportion of the total population considered trust in healthcare as a key promoter compared to the vaccinated sample (62.0%). There was little difference between the vaccinated and total populations for the remainder of the top-five promoters, where the availability and/or time constraints of participants were the second most agreed upon barrier, as reported by 57.0% of participants for both groups. Knowledge of vaccination was found to be equal across both participant groups. Costs, including all questions investigating direct, indirect, or resource utilization costs, were not among the top-five most agreed upon promoters for vaccination against influenza.

Availability was an equally important promoter of vaccination for both the total and vaccinated populations. An unprompted comment about the importance of availability in intention to vaccinate from the Quinn et al. 2016 trial stated “It’s a pain organizing a trip to the doctor. Having it be very convenient makes it easy.” [50]. Another vaccinated participant from the same study explained the many facets of trust involved with vaccination; “I trust that the vaccine is going to be effective, I trust that nothing dangerous is being given to me, and I trust the sources of the vaccine, I’m trusting the makers of the vaccine, I’m trusting my doctor who recommends it, and I’m trusting the U.S. government who promotes it. So, it is a lot of trust.” [50]. This statement highlights the importance of factoring in all stages of healthcare when considering trust in healthcare as a promoter of vaccination.

### 3.6. Intent to Vaccinate

Intent to vaccinate against influenza in the current or following season was reported by 34 publications, 17 of which reported data on participants willing to be vaccinated, whereas 19 reported on participants unwilling to be vaccinated (2 publications reported data for participants willing and unwilling to be vaccinated) [18,19,20,21,23,24,25,28,29,34,37,39,40,42,44,48,49,51,53,61,71,73,75,83,85,86,87,88,89,90,91,92,93,94]. Of the 17 publications investigating participant’s willingness to be vaccinated, 44.6% of participants agreed that they would be willing to vaccinate against influenza in the current or following season. Conversely, in the 19 studies that investigated participants unwilling to be vaccinated, 47.1% of participants reported that they were unwilling to be vaccinated in the current or following season. Studies investigating willingness to be vaccinated could not be combined with those investigating unwillingness to be vaccinated due to the heterogeneity between question styles asked of the participants.

Participants who were male and/or listed their previous vaccination history appeared to be positively associated with a greater intention to be vaccinated against influenza in the future. Impact of sex on decision to be vaccinated was assessed in eight studies; three studies investigated differences between participants willing to be vaccinated; and five studies reported on participants unwillingness to be vaccinated. There was a slightly higher agreement with willingness to be vaccinated observed in males (45.6%) compared to females (43.6%) [25,51,85]. This trend was further supported by a lower number of male participants being unwilling to be vaccinated (43.5%) than females (52.4%) [25,85,86,90,91]. Five studies reported the difference in willingness to be vaccinated between participants with a previous history of vaccination. Vaccinated participants demonstrated an increased intent to be vaccinated against influenza (81.5%) than unvaccinated participants (59.1%) [19,21,37,42,51]. There were lower rates of agreement when participants were asked if they were unwilling to be vaccinated against influenza, where 39.9% of unvaccinated participants and only 7.8% of previously vaccinated participants would be unwilling to be vaccinated in the current or following season [20,23,24,28,34,75,90].

The Werneck et al. 2021 study was one of two studies identified by this review that investigated racial differences between participants unwilling to be vaccinated in the future. It was found that Brazilian civil servants, no matter their race, showed comparable intent towards the influenza vaccine, whereas approximately 16% were not willing to be vaccinated [91]. The second study, Crouse-Quinn et al. 2017, conducted in the US, reported that significantly fewer high-risk black participants (those with chronic comorbidities, which would be exacerbated by influenza) were willing to immunize against influenza than white participants with comorbidities, suggesting racial disparity in vaccine uptake [40].

Sixteen studies reported on data collected from 2020 onwards, of which a single longitudinal study investigated a change in knowledge, attitudes, and intent to be vaccinated during the COVID-19 pandemic [17,19,20,25,28,36,38,49,61,62,71,78,86,87,88,92]. Domnich et al. 2021 combined two cross-sectional questionnaires from 2020 and 2021 to compare the change in attitudes towards the influenza vaccine [36]. The study population consisted of 2,543 Italian adults (≥18 years), of which the mean age was 48.3 years and 45.1% were female [36]. There was a significant increase in trust of vaccines from 18.3% to 25.6% (*p* < 0.001) as the COVID-19 pandemic continued, as more people agreed that influenza vaccination should be mandatory and less believed it was a “fraud” [36]. In 2021, both knowledge and interest in knowledge acquisition had increased from 2020. Despite a significant portion of the Italian population remaining hesitant towards vaccination, public confidence in vaccinations increased significantly (*p* < 0.001). As reported by the publication, this was “at least partially determined by the ongoing COVID-19 pandemic” [36].

### 3.7. Strategies to Overcome Barriers

**Figure 5:**
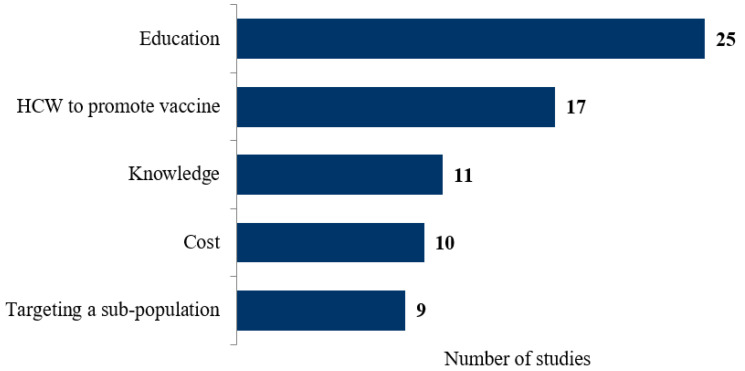
Strategies to overcome barriers to influenza vaccination. HCW: Healthcare worker.

Of the 80 publications included in this review, 64 suggested strategies to overcome barriers and increase uptake of the influenza vaccine [16,17,18,19,20,21,22,23,24,25,27,28,29,30,31,32,33,34,35,38,39,40,41,42,44,45,46,47,48,49,50,51,52,53,54,55,56,58,59,60,61,62,64,65,67,68,69,70,72,73,75,76,78,79,80,81,82,83,84,85,87,89,92,93,95]. The strategies presented in Figure 5 focus on the five key emerging themes extracted from the author recommendations. These strategies were not mutually exclusive, as some authors recommended several strategies. Education was the most common strategy proposed to improve vaccination uptake, as reported by 25 studies. Other key themes reported by the included publications were communication (N = 7), awareness (N = 7), accessibility (N = 4), importance (N = 2), and policy update (N = 1). Examples of the most common strategies reported by publications are provided in Table 6.

## 4. Discussion

Influenza is associated with a significant global clinical, humanistic, and economic burden [5,96]. Vaccines are key to the control of the influenza virus; however, despite recommendations from governing public-health bodies, there remains considerable variation in vaccination coverage rates globally [8]. To elucidate reasons for low vaccination coverage rates, this review aimed to understand the attitudes and perceived barriers of the adult general population toward influenza vaccine uptake from a global viewpoint. Data identified in this review suggested that approximately 50% of the adults surveyed intended to receive the vaccination in the following/upcoming influenza season [18,19,20,21,23,24,25,28,29,34,37,39,40,42,44,48,49,51,53,61,71,73,75,83,85,86,87,88,89,90,91,92,93,94]. The most frequently agreed upon barrier to uptake was a perceived lack of knowledge of the influenza vaccine, while trust in healthcare services was the most agreed upon promoter for vaccine uptake. The findings of this review suggest that strategies to improve vaccine uptake should focus on education to improve public knowledge of influenza vaccines and the value of vaccination. These educational materials should be utilized by trusted HCWs to maximize impact.

This review found that a negative attitude towards healthcare was the most commonly agreed upon barrier to vaccination against influenza among the adult general population, which reflects findings from previously conducted literature [10,97]. Among unvaccinated participants specifically, the barrier to uptake with the highest agreement rate was a perceived lack of knowledge, where participants described a lack of knowledge across the entire vaccination process [50]. These review findings are not fully aligned with the most widely reported barrier to vaccination against influenza for the adult general population in the literature, which was the fear of vaccine-associated side effects [10,98,99]. However, a fear of vaccination-associated side effects may lead to participants stating poor knowledge of vaccine safety and delaying or refusing vaccination, suggesting a potential correlation between these findings. Further educational materials detailing the safety and efficacy of influenza vaccines may provide the necessary reassurance for the adult general population and improve vaccination uptake [18,38,47,54,55,56,73,89,95].

Of the adult general population, some subgroups of participants appeared more likely to be vaccinated against influenza than others. The findings from this review echo previously published literature, as there appeared to be an association between previous vaccination history and increased uptake rates [100,101]. This suggests that previous experience with influenza vaccination has a substantial influence on willingness to be vaccinated in the upcoming vaccination season. Two publications identified in this review reported intention to be vaccinated by race. Werneck et al. 2021 found no significant difference between white and black/Hispanic Brazilian civil servants not willing to be vaccinated against influenza in the upcoming season, while Crouse-Quinn et al. 2017 reported that significantly fewer high-risk black participants were immunizing than high-risk white participants [40,91]. The latter finding was consistent with the published literature, as multiple studies have identified a significantly reduced likelihood to be vaccinated against influenza among black and Hispanic populations compared to white people [13,102]. The difference between these two findings may be due to the Werneck et al. 2021 study, including exclusively civil servants, unlike the adult general population captured by Crouse-Quinn et al. 2017 [40,91]. Similar observations of racial and socioeconomic disparities in vaccine uptake have been reported for other vaccines, such as HPV, pneumococcal, and meningococcal vaccines [103,104,105].

Sex differences were found in a small number of publications included in this review; however, comparison between demographic groups, such as age, income, education status, employment, and comorbidity status, was limited due to study population heterogeneity. Future studies investigating comparisons between these demographics over a longitudinal study program may help to elucidate key sub-populations to target with educational materials.

Costs were not reported to be amongst the top-three barriers identified in this review; however, it should be considered that the included countries have varying reimbursement strategies for different patient groups and, hence, responses towards costs will vary greatly between regions [21,24,27,33,39,52,67,69,73,82]. Costs could become a more significant barrier to influenza vaccination once the knowledge barrier has been overcome, specifically in those countries with less supportive reimbursement strategies.

The findings from this review suggest that doubts prevail in a large proportion of the adult general population regarding the perceived benefit of influenza vaccination [18,35,56]. Depending on geographical location and guidelines, adults aged 18–64 may not be prioritized to receive influenza vaccines. Despite recommendations, vaccinating this population has a substantial impact on preventing the spread of influenza to older adults (>65 years) and pediatric populations and should be taken into consideration [106,107]. From a wide geographical perspective, it was noted that healthcare systems should aim to overcome this attitude towards vaccination by encouraging the promotion of influenza vaccination by healthcare professionals, increasing the volume of education programs, and improving public knowledge of vaccination safety and efficacy [16,17,18,19,20,21,22,23,24,25,27,28,29,30,31,32,33,34,35,38,39,40,41,42,44,45,46,47,48,49,50,51,52,53,54,55,56,58,59,60,61,62,64,65,67,68,69,70,72,73,75,76,78,79,80,81,82,83,84,85,87,89,92,93]. To maximize impact, educational materials should include clear and concise fact-based messages highlighting the benefits of vaccination against influenza before directly addressing common myths and misconceptions [108]. Closing the knowledge gap may be achieved by promotion of materials by HCWs, as demonstrated by the study by Gorman et al. 2012, which found that women were up to three-times more likely to seek vaccination following encouragement by HCWs [109]; hence, ensuring HCWs are participating in an active dialogue, encouraging vaccination for all patients, could improve overall vaccine uptake [99].

In order to fully understand the key reasons underlying intention to be vaccinated, this review captured the positive attitudes towards influenza vaccination reported in the included studies. Trust in healthcare was observed to be the greatest promoter of vaccination for the total cohort and even greater among the vaccinated population. This review supports the notion that improving trust should be at the forefront of influenza vaccine implementation research, where increasing trust in a wider population would result in improved overall vaccine coverage [40]. Improving vaccine effectiveness would likely be an effective way to support education materials; hence, novel approaches to vaccination strategies, such as messenger ribonucleic acid (mRNA) vaccines, could improve global trust in vaccine effectiveness, which would, in turn, improve trust in healthcare systems and ultimately improve vaccine uptake.

The COVID-19 pandemic has revitalized public and media interest in vaccination. The single longitudinal study identified in this review that surveyed participants during the first and second year of the COVID-19 pandemic (2020–2021) reported a significant increase in trust of vaccines, along with an increased demand for further information on influenza vaccines [36]. Other recent studies reported a similar finding, where intent to be vaccinated against influenza was reported to have increased during the COVID-19 pandemic [110,111,112]. Del Riccio et al. 2021 reported the influenza vaccine coverage rates across 10 northern hemisphere countries (England, France, Israel, Italy, The Netherlands, Philippines, Poland, South Korea, Spain, United States) and in Australia. Except for South Korea, all countries reported an increase in vaccine coverage rate from 2019/2020 to the 2020/2021 season (range: +3.0–13.0%) [113]. The coverage rates pre-pandemic and during the pandemic reiterate the previously reported increase in trust and demand for influenza vaccines as a result of COVID-19. There remains a scarcity of evidence investigating the cause of this, as multiple factors influence vaccination intent and are highly likely to vary depending on a multitude of situational factors [10,58,114].

It has been reported that significant hesitancy towards the influenza vaccine still exists, due to perceived low risk of illness combined with safety and efficacy concerns [110]. Egg-derived influenza vaccines are the most distributed influenza vaccines globally [115]. However, these traditional vaccines display suboptimal vaccine effectiveness, in terms of strength and longevity of immunogenicity, resulting in a limited breadth of protection across influenza strains [116]. Recent technological advances have helped to provide a promising improvement in the rapid production of mRNA vaccines that have the capacity for high potency at a low overall manufacturing cost [117]. Using mRNA technology in the development of influenza vaccines could, therefore, be a promising approach to improve future uptake rates and trust in healthcare. The increased demand for knowledge of the safety and efficacy of influenza vaccines needs to be addressed by healthcare systems worldwide, perhaps with lay education on novel vaccine production methods such as these.

### Strengths and Limitations

The primary limitation of this review was the large heterogeneity in question types asked by the included studies, resulting in challenges in grouping the data consistently. Question types were interpreted by the reviewers and, despite clear definitions (Table 2), inadvertently were subject to bias and varying interpretations between reviewers. The inclusion criteria specified outcomes based on patient perspectives; hence, most of the included publications followed a cross-sectional study design, which may have limited information on the type of vaccination received by participants. Vaccination status was self-reported in many studies and sampling methods were subject to selection bias in some publications. This approach did, however, allow for a large number of studies reporting directly on participant attitudes towards influenza vaccinations to be captured. Searches for this review were restricted to English language only, although many publications were included from non-English-speaking countries. Despite this review capturing a substantial adult population from multiple countries (N = 257,202 participants), studies included were largely based in North America and Western European countries and may not be reflective of the attitudes and barriers globally or in geographies not captured by this review. This population will, however, have large variations in reimbursement strategies, vaccination guidelines/availability, and healthcare systems, resulting in findings that may not be representative of individual countries or specific regions. The many strategies identified by this review to overcome barriers to influenza vaccination identify multiple tactics for healthcare agencies globally to improve vaccine uptake.

## 5. Conclusions

The evidence captured by this review suggests that the main barriers to influenza vaccine uptake are a combination of limited vaccine knowledge and negative attitudes towards healthcare services. Conversely, the promoter of vaccine uptake with the highest agreement rate was trust in healthcare. Several identified studies recommended that improved education regarding the safety and efficacy of influenza vaccination would improve uptake in the adult general population. To act on this, healthcare systems should arm HCWs with clear and concise evidence to educate patients. Over the past few years, the COVID-19 pandemic thrust vaccines back into the public eye, with particular attention on the rapid development of the novel mRNA vaccines that are exquisitely responsive to epidemiologic changes. Improved vaccines and further education on the benefits of vaccine uptake may help to overcome the identified barriers and may ultimately improve vaccine coverage rates for influenza.

## Figures and Tables

**Table 1:** Eligibility criteria.

Topic	Inclusion Criteria	Exclusion Criteria
**Population(s)**	Adults (18–64 years) eligible for the influenza vaccine in the following countries: France Germany Italy Spain United Kingdom United States Canada Mexico China Japan Brazil Saudi Arabia South Africa Taiwan Hong Kong Australia	Any other region/country. Focus on pediatric or elderly (≥65 years) populations. Focus on pregnant population. Studies reporting primarily on healthcare workers.
**Interventions**	Any the following seasonal influenza vaccinations: Recombinant vaccine Trivalent vaccine Quadrivalent vaccine Inactivated vaccine Live attenuated vaccine mRNA vaccine Intramuscular vaccine Intranasal vaccine Intradermal vaccine	N/A
**Comparisons**	Any/none	N/A
**Outcomes**	Data on barriers or attitudes to influenza uptake and/or strategies to improve uptake, including: Patient attitudes and perceptions towards vaccine (safety, efficacy) and healthcare system/professionals. Outcomes from a patient perspective Attitude towards vaccine technology. Accessibility and availability of vaccine. Vaccine hesitancy including altered vaccine schedule, or delayed acceptance. Perceived barriers (contextual, social, psychological). Barriers to seasonal influenza vaccines compared to non-seasonal vaccines (e.g., Human papillomavirus, Varicella, etc.). Direct and indirect costs as a barrier to uptake. Refusal rate. Intention to vaccinate self and/or dependents (i.e., children). Satisfaction level with vaccine. Preference to receive COVID-19 vaccine over influenza vaccine. Impact of previous vaccination experience on future uptake.	Studies not reporting barriers or attitudes towards influenza vaccinations. Studies reporting only on uptake rate with no mention of barriers/attitudes. Not reporting outcomes from patient perspective.
**Time**	Published from January 2012 to 6 May 2022.	N/A
**Study design**	Clinical studies Case control studies Cohort studies Observational studies Longitudinal studies Epidemiological studies Cross-sectional studies Systematic literature reviews Meta-analyses Real world evidence/data Database studies (medical records, claims) Patient/physician surveys/questionnaires Patient/physician preference studies	Randomized controlled trials Editorials Letters to journals Non-systematic literature reviews Conference minutes
**Other**	Human studies English language	Animal studies Duplicates Non-English language

mRNA: messenger ribonucleic acid; N/A: Not applicable.

**Table 2:** Categorization of question types.

Category	Question Type	Definition	Barrier Example	Promoter Example
**Access**	Access to vaccine	Questions that determine the access that a participant has to influenza vaccination	“Getting the flu vaccine required a lot of effort on my part” [16]	“Ease of access” [17]
**Access**	Availability/time constraints	Questions that refer to the participant’s availability to obtain the influenza vaccination	“I don’t have the time to get vaccinated” [18]	“Supposed to receive vaccination in the workplace” [19]
**Access**	Recommended by HCW	Questions investigating influence of recommendation by HCWs	N/A	“The specialists encouraged a vaccination.” [20]
**Access**	Transport	Questions that determine any transportation restraints of the participants	“Inconvenient to reach a vaccination location” [21]	“Convenient to reach a vaccination location” [21]
**Cost**	Cost	Questions that highlight cost as the determining factor over choice of vaccination	“Could not afford vaccination” [22]	“Vaccine was a reasonable price” [23]
**Intent to vaccinate**	Intent to vaccinate	Intent of the participant to vaccinate in the upcoming influenza season(s)	“Unwilling to receive influenza vaccination” [24]	“Willingness to Receive Influenza Vaccination” [25]
**Knowledge**	Knowledge	Questions referring to the knowledge that the participant has of influenza vaccinations	“Believed the vaccine causes influenza” [26]	“Even if infected with influenza, wanted to prevent the symptoms from becoming serious” [22]
**Non-optional**	Health exemptions	Avoidance of vaccination due to medical reasons (such as allergies)	“I am allergic to flu vaccine” [27]	N/A
**Non-optional**	Requirement (for job/religion)	Requirement of vaccination (by either employer or religion)	N/A	“It is mandatory for my work” [28] “Hajj requirement” [29]
**Psychological**	Past behaviors	Questions relating to the behavior of participants towards vaccines in previous years	“Previously rejected influenza vaccine” [30]	“I am accustomed to getting a flu shot each year” [31]
**Psychological**	Past experiences	Questions investigating the effect of past experiences with vaccination	“Bad reaction to previous shot” [32]	“Suffered from influenza last year” [33]
**Social**	Attitude towards healthcare	Questions that determine the attitude of the participant towards healthcare	“Perception of low self-risk” [34]	“The best way to avoid the complications of influenza is by using influenza vaccine” [35]
**Social**	Subjective beliefs	Questions used to determine the beliefs of a participant in relation to the influenza vaccine	“I think it is harmful” [17]	“Vaccines are crucial to guaranteeing public health and should be mandatory” [36]
**Trust**	Trust in healthcare	Questions used to determine the trust in healthcare and government guidelines that the participant has	“I don’t trust vaccines” [37]	“Influenza vaccine is safe and effective” [35]

HCW: Healthcare worker; N/A: Not applicable.

**Table 3:** Participant demographics reported by US studies (N = 22) [16,26,28,31,34,38,39,40,41,42,43,44,45,46,47,48,49,50,51,52,53,54].

Participant Demographic	US Results
**Sample size, mean**	673
**Age, mean (range)**	44.4 years (15–94)
**Sex: Female**	54.8%
**Race/ethnicity (verbatim from text)**	Black, White, Asian, South Asian, Hispanic or Latino, American Indian or Alaska Native, Native Hawaiian/other Pacific Islander, Mixed Race.
**Employment status**	Full-time, part-time, unemployed, retired, student, disabled.
**Income range (USD)**	<15,000 to ≥150,000
**Undergraduate education or above**	62.9%
**Comorbidities (verbatim from text)**	High-risk population, psychiatric patients, cardiovascular disease, rheumatoid arthritis, pulmonary disease, asthma, chronic bronchitis, cancer (all types except for skin cancer), cystic fibrosis, diabetes, epilepsy, heart attack, heart disease, high blood pressure, HIV/AIDS, kidney disease, stroke, renal dysfunction, hemoglobinopathy, immunosuppression.
**Current season vaccination rate**	43.7%

AIDS: Acquired immunodeficiency syndrome; HIV: Human immunodeficiency virus; US: United States; USD United States dollar.

**Table 4:** Prevalence of barrier types reported by the included studies.

Barrier	Number of Questions Investigating Barrier		Agreement Rate of Participant with Barrier (%)	
	Total Population	Unvaccinated	Total Population	Unvaccinated
**Trust**	98	26	20.6	14.1
**Knowledge**	40	6	19.3	32.3
**Costs**	28	10	15.5	27.3
**Social**	276	67	14.1	14.5
**Psychological**	89	16	13.0	22.0
**Access**	105	29	10.0	12.7
**Health**	21	10	1.8	2.2

**Table 5:** Prevalence of promoter types reported by the included studies.

Promoter	Number of Questions Investigating Promoter		Agreement Rate of Participant with Promoter (%)	
	Total Population	Vaccinated	Total Population	Vaccinated
**Trust**	91	8	68.1	79.0
**Social**	125	26	47.6	45.5
**Costs**	15	4	44.1	41.1
**Knowledge**	22	5	43.8	51.5
**Access**	28	14	31.8	26.1
**Non-optional**	7	3	21.1	10.9
**Psychological**	14	1	20.3	5.4

**Table 6:** Strategies to improve influenza vaccination rate.

Strategy to Improve Influenza Vaccination Rate	Example
**Education**	“Increased efforts to educate college students about the risks and importance of the vaccine may serve to minimize widely held misconceptions about the vaccine.” [79].
**HCW to promote vaccination**	“Primary care physicians should intensively promote vaccination because vaccination recommendation by a physician and information dissemination regarding vaccines and vaccination to patients significantly increase vaccination rates.” [29].
**Improving public knowledge**	“False fear from vaccine complications is by far the most significant and the one that requires urgent attention.” [76].
**Costs**	“There is need for a different government approach to resolving the financial deficit in Italy focused on health promotion and disease prevention.” [24].
**Targeting a specific sup-population**	“Information strategies and vaccination campaigns need to be adapted to the characteristics of the targeted population.” [59].

HCW: Healthcare worker.

## Data Availability

No new data were created or analyzed in this study. Data sharing is not applicable to this article.

## Supplementary Materials

The following supporting information can be downloaded at: https://www.mdpi.com/article/10.3390/vaccines11010180/s1, Table S1: Embase search strategy; Table S2: Medline search strategy; Table S3: Quality appraisal checklist.
