# Supplementary material for: Understanding the Barriers and Attitudes toward Influenza Vaccine Uptake in the Adult General Population: A Rapid Review
Source: Vaccines (Basel). 2023 Jan 13;11(1):180. doi: 10.3390/vaccines11010180 (PMC9861815; doi:10.3390/vaccines11010180)
Supplement: Supplementary file 1 [file vaccines-11-00180-s001.zip › vaccines-2117615-supplementary.pdf]

Supplementary material

Search strategies

Table S1. Embase search strategy

#		Searches	Results
1	Disease terms	exp *influenza vaccine/	19,034
2		exp *influenza/	49,024
3		exp *Influenza A virus/ or exp *Influenza B virus/	7,481
4		(flu or influenza).ti,ab.	125,478
5		or/1-4	129,939
6	Intervention terms	exp recombinant vaccine/ or exp live vaccine/ or exp virus vaccine/ or exp vaccine/ or exp subunit vaccine/ or exp DNA vaccine/ or exp acellular vaccine/ or exp virosome vaccine/ or exp inactivated vaccine/ or RNA vaccine/ or "mRNA vaccine".ti,ab.	340,195
7		exp immunization/	295,367
8		exp vaccination/	185,875
9		exp active immunization/	6,324
10		exp immunoprophylaxis/	4,667
11		exp mass immunization/	4,171
12		(vaccin* or immuni* or inocul*).ti,ab.	764,131
13		or/6-12	878,351
14	Outcome terms	(barrier* or uptake or refus* or decline*).ti,ab.	1,325,083
15		"patient attitude"/ or (patient$ adj2 (attitude$ or compliance or "non compliance" or adheren$ or "non adherence" or participation or "non participation" or preference$ or satisf$ or dissatisf$ or toleran$ or intoleran$ or "reported outcome" or "reported outcomes")).ti,ab.	289,995
16		vaccine hesitancy/	2,837
17		hesitan*.ti,ab.	7,254
18		(preference* adj3 (valu* or measur* or health or life or estimat* or elicit* or disease or score* or instrument or instruments)).ti,ab.	16,448
19		("vaccin* acceptance" or "delayed vaccin* acceptance" or "altered vaccin* schedule").ti,ab.	1,492
20		((vaccin* or immuni* or inocul*) adj5 (belief* or doubt* or fear* or hesitanc* or trust or mistrust or delay or reject* or refus*)).ti,ab.	9,391
21		((patient or physician) adj3 (accept* or willingness)).ti,ab.	13,649
22		or/14-21	1,618,838
23	Study design terms	Clinical study/	109,621
24		exp case control study/	204,949
25		Family study/	24,969
26		Longitudinal study/	169,214
27		Retrospective study/	1,227,879

28		Prospective study/	756,324
29		Cohort analysis/	831,857
30		(Cohort adj (study or studies)).mp.	396,336
31		(Case control adj (study or studies)).tw.	151,338
32		(follow up adj (study or studies)).tw.	61,725
33		(observational adj (study or studies)).tw.	214,400
34		(epidemiologic$ adj (study or studies)).tw.	108,351
35		(cross sectional adj (study or studies)).tw.	284,389
36		exp observational study/	269,991
37		exp cross-sectional study/	475,391
38		exp medical record/	279,087
39		exp "administrative claims (health care)"/	1,000
40		((longitudinal* or retrospective* or prospective* or observ* or cohort or case control or cross sectional or follow up or pragmatic or claims or routine care) adj stud*).mp.	3,226,065
41		(real world adj (data or stud* or effect* or evidence)).mp.	25,817
42		((database or medical record*) adj3 (review or stud*)).mp.	194,705
43		((semi structured or unstructured or structured) adj2 (interview* or survey* or focus group* or questionnaire*)).ti,ab.	122,584
44		exp Meta Analysis/ or ((meta adj analys*) or metaanalys*).ti,ab.	342,542
45		"systematic review"/ or ("systematic literature review" or "SLR").ti,ab.	349,993
46		or/23-45	4,432,944
47		(exp Animal/ or nonhuman/) not exp human/	5,642,172
48		exp case study/ or exp case report/ or exp letter/ or exp preliminary communication/ or exp note/ or exp editorial/ or exp editor/ or exp editorial policies/ or exp newspaper/	4,766,873
49		or/47-48	10,267,934
50		46 not 49	4,105,493
51	Total	5 and 13 and 22 and 50	2,210
52		limit 51 to yr="2012 -Current"	1,848

Table S2. Medline search strategy

#		Searches	Results
1	Disease terms	exp Influenza, Human/	54,714
2		exp Influenza Vaccines/	25,450
3		exp Orthomyxoviridae/	61,817
4		(flu or influenza).ti,ab.	116,270
5		or/1-4	128,605
6	Intervention terms	exp Vaccines, Synthetic/	30,996
7		exp Viral Vaccines/	130,757
8		exp Vaccines, Attenuated/	12,742

9		exp Vaccines, Subunit/	6,162
10		exp Vaccines, DNA/	10,515
11		exp Vaccines, Acellular/	1,678
12		exp Vaccines, Virosome/	224
13		exp Vaccines, Inactivated/	9,022
14		exp mRNA Vaccines/	2,059
15		exp Immunization/	196,620
16		exp Mass Vaccination/ or exp Anti-Vaccination Movement/ or exp Vaccination/	99,408
17		exp Vaccination Hesitancy/ or exp Vaccination Coverage/ or exp Vaccination Refusal/	3,127
18		exp Immunization/ or exp Immunization Programs/	202,861
19		(vaccin* or immuni* or inocul*).ti,ab.	738,256
20		or/6-19	810,753
21	Outcome terms	(barrier* or uptake or refus* or decline*).ti,ab.	1,119,805
22		(patient$ adj2 (attitude$ or compliance or "non compliance" or adheren$ or "non adherence" or participation or "non participation" or preference$ or satisf$ or dissatisf$ or toleran$ or intoleran$ or "reported outcome" or "reported outcomes")).ti,ab.	146,665
23		(hesitan* or "vaccin* acceptance" or "delayed vaccin* acceptance" or "altered vaccin* schedule").ti,ab.	6,813
24		(preference* adj3 (valu* or measur* or health or life or estimat* or elicit* or disease or score* or instrument or instruments)).ti,ab.	12,868
25		((vaccin* or immuni* or inocul*) adj5 (belief* or doubt* or fear* or hesitanc* or trust or mistrust or delay or reject* or refus*)).ti,ab.	8,329
26		((patient or physician) adj3 (accept* or willingness)).ti,ab.	9,681
27		or/21-26	1,281,156
28	Study design terms	Clinical Study/	4,710
29		Case-Control Studies/	320,643
30		Longitudinal Studies/	157,536
31		Retrospective Studies/	1,021,428
32		Prospective Studies/	625,406
33		Cohort Studies/	311,668
34		((Cohort or "case control" or "follow up" or observational or "cross sectional") adj (study or studies)).mp.	2,034,036
35		Observational Study/	126,159
36		Cross-Sectional Studies/	423,044
37		((longitudinal* or retrospective* or prospective* or observ* or cohort or case control or cross sectional or follow up or pragmatic or claims or routine care) adj stud*).mp.	3,303,799
38		(real world adj (data or stud* or effect* or evidence)).mp.	12,738
39		Medical Records/	66,103
40		((database or medical record*) adj3 (review or stud*)).mp.	32,620
41		((semi structured or unstructured or structured) adj2 (interview* or survey* or focus group* or questionnaire*)).ti,ab.	93,474

42		exp Meta Analysis/ or ((meta adj analys*) or metaanalys*).ti,ab. or "systematic review"/ or ("systematic literature review" or "SLR").ti,ab.	352,607
43		or/28-42	3,723,489
44		(exp Animal/ or nonhuman/) not exp human/	4,998,985
45		exp case study/ or exp case report/ or exp letter/ or exp preliminary communication/ or exp note/ or exp editorial/ or exp editor/ or exp editorial policies/ or exp newspaper/	3,825,013
46		5 and 20 and 27 and 43	1,661
47		46 not (or/44-45)	1,655
48	Total	limit 47 to yr="2015 -Current"	1,060

Study quality appraisal checklist

Table S3. Quality appraisal checklist

**Study name**<br>**Study question**	**Response (Yes/No/N/A)**
Was the sample frame appropriate to address the target population?	
Were study participants sampled in an appropriate way?	
Was the sample size adequate?	
Were the study subjects and the setting described in detail?	
Was the data analysis conducted with sufficient coverage of the identified sample?	
Were valid methods used for the confirmation of vaccination?	
Was vaccination confirmation measured in a standard, reliable way for all participants?	
Was there appropriate statistical analysis?	
Was the response rate adequate, and if not, was the low response rate managed appropriately?	

Adapted from “JBI Manual for Evidence Synthesis”[12]
JBI: Joanna-Briggs Institute, N/A: Not Applicable.

Table S4. JBI quality appraisal of included studies

Study	Was the sample frame appropriate to address the target population?	Were study participants sampled in an appropriate way?	Was the sample size adequate?	Were the study subjects and the setting described in detail?	Was the data analysis conducted with sufficient coverage of the identified sample?	Were valid methods used for the confirmation of vaccination?	Was vaccination confirmation measured in a standard, reliable way for all participants?	Was there appropriate statistical analysis?	Was the response rate adequate, and if not, was the low response rate managed appropriately?	Overall appraisal
Mongeau et al. 2022										Low risk of bias
Barry et al. 2020										High risk of bias
Aljamili et al. 2020										High risk of bias
Almotairy et al. 2019										Unclear
Genovese et al. 2022										High risk of bias
Liu et al. 2022										Low risk of bias
Kosaka et al. 2021										High risk of bias
Gong et al. 2021										Low risk of bias
Domnich et al. 2021										Low risk of bias
Dallagiacoma et al. 2021										Low risk of bias
Johnson et al. 2021										Low risk of bias
Alhawsawi et al. 2020										Low risk of bias
Kawahara et al. 2020										High risk of bias
Werneck et al. 2021										Low risk of bias

Trent et al. 2020										High risk of bias
Al Hassan et al. 2021										Low risk of bias
Mercadante et al. 2021										Low risk of bias
Yan et al. 2021										High risk of bias
Figueroa-Parra et al. 2021										High risk of bias
Chen et al. 2020										Low risk of bias
Maguire et al. 2019										Low risk of bias
Lejri-El Euchi et al. 2019										Low risk of bias
Feng et al. 2019										Low risk of bias
Luz et al. 2019										High risk of bias
Guay et al. 2019										Low risk of bias
Bertoldo et al. 2019										Low risk of bias
Jiang et al. 2019										Low risk of bias
Roy et al. 2018										Low risk of bias
Sagor et al. 2018										Low risk of bias
Casalino et al. 2018										High risk of bias
Wu et al. 2018										Low risk of bias
Fall et al. 2018										High risk of bias
Alabbad et al. 2018										Low risk of bias
Cotugno et al. 2017										Low risk of bias

Crouse Quinn et al. 2017										High risk of bias
Olatunbosun et al. 2017										High risk of bias
Luz et al. 2017										High risk of bias
Wheelock et al. 2017										Low risk of bias
Jimenez-Garcia et al. 2017										High risk of bias
Hilger et al. 2016										High risk of bias
Wong et al. 2016										Low risk of bias
Yeung et al. 2015										High risk of bias
Gilstad-Hayden et al. 2015										High risk of bias
Quinn et al. 2016										High risk of bias
Murray et al. 2016										High risk of bias
Valerio et al. 2022										High risk of bias
Domnich et al. 2022										High risk of bias
Montalti et al. 2021										High risk of bias
Harada et al. 2022										High risk of bias
Fayed et al. 2021										High risk of bias
Tao et al. 2021										High risk of bias
Chu et al. 2021										Low risk of bias
Lytle et al. 2021										High risk of bias
Domnich et al. 2020										High risk of bias

Alzeer et al. 2021										High risk of bias
La Vecchia et al. 2020										High risk of bias
Bauer et al. 2020										Low risk of bias
Rempenault et al. 2020										High risk of bias
Quezada et al. 2019										High risk of bias
Soler Hildago et al. 2019										Unclear
Nestor et al. 2019										High risk of bias
Ntatsaki et al. 2018										High risk of bias
Mendez-Roldan et al. 2018										Unclear
Khalid et al. 2018										High risk of bias
Benjamin et al. 2016										High risk of bias
Nunley et al. 2016										High risk of bias
Sandler et al. 2014										High risk of bias
de Perio et al. 2014										High risk of bias
Radtke et al. 2013										Low risk of bias
Wada et al. 2013										High risk of bias
Cohen et al. 2013										High risk of bias
Uppal et al. 2013										High risk of bias
Iwasa et al. 2013										Low risk of bias
Schlenker et al. 2013										Unclear

Wu et al. 2013										Low risk of bias
Rodas et al. 2012										High risk of bias
Blank et al. 2012										Low risk of bias
de Souza et al. 2012										Low risk of bias
Gidengil et al. 2012										Low risk of bias
Böhmer et al. 2012										Low risk of bias

	Respond “yes” to question, low risk of bias
	Respond “no” to question, high risk of bias
	Insufficient/unclear evidence to answer question, unclear level of bias
	Not applicable
